# The Phenotype Changes of Astrocyte During Different Ischemia Conditions

**DOI:** 10.3390/brainsci14121256

**Published:** 2024-12-14

**Authors:** Fei Meng, Jing Cui, Peng Wang, Junhui Wang, Jing Sun, Liang Li

**Affiliations:** 1 Cardiac Valve Center, Department of Cardiac Surgery, Beijing Anzhen Hospital, Capital Medical University, Beijing 101100, China; mengfei_117@sohu.com; 2 Department of Pathology, School of Basic Medical Sciences, Capital Medical University, Beijing 100069, China; cuijing1212@163.com (J.C.); sunjing@ccmu.edu.cn (J.S.); 3 The Key Laboratory of Cardiovascular Remodeling and Function Research, Chinese Ministry of Education and Chinese Ministry of Public Health, Department of Cardiology, Qilu Hospital, Shandong University, Jinan 250012, China; 17866806032@163.com; 4 Lunenfeld-Tanenbaum Research Institute, Mount Sinai Hospital, Toronto, ON M5G 1X5, Canada; junhui@lunenfeld.ca

**Keywords:** dementia, ischemia, astrocytes, A2 phenotype, Connexin 43

## Abstract

Objectives: Dementia is becoming a major health problem in the world, and chronic brain ischemia is an established important risk factor in predisposing this disease. Astrocytes, as one major part of the blood–brain barrier (BBB), are activated during chronic cerebral blood flow hypoperfusion. Reactive astrocytes have been classified into phenotype pro-inflammatory type A1 or neuroprotective type A2. However, the specific subtype change of astrocyte and the mechanisms of chronic brain ischemia are still unknown. Methods: In order to depict the phenotype changes and their possible roles during this process, a rat bilateral common carotid artery occlusion model (BCAO) was employed in the present study. Meanwhile, the signaling pathways that possibly regulate these changes were investigated as well. Results: After four-week occlusion, astrocytes in the cortex of BCAO rats were shown to be the A2 phenotype, identified by the significant up-regulation of S100a10 accompanied by the down-regulation of Connexin 43 (CX43) protein. Next, we established in vitro hypoxia models, which were set up by stimulating primary astrocyte cultures from rat cortex with cobalt chloride, low glucose, or/and fibrinogen. Consistent with in vivo data, the cultured astrocytes also transformed into the A2 phenotype with the up-regulation of S100a10 and the down-regulation of CX43. In order to explore the mechanism of CX43 protein changes, C6 astrocyte cells were handled in both hypoxia and low-glucose stimulus, in which decreased pERK and pJNK expression were found. Conclusions: In conclusion, our data suggest that in chronic cerebral ischemia conditions, the gradual ischemic insults could promote the transformation of astrocytes into A2 type instead of A1 type, and the phosphorylation of CX43 was negatively regulated by the phosphorylation of ERK and JNK. Also, our data could provide some new evidence of how to leverage the endogenous astrocytes phenotype changes during CNS injury by promoting them to be “protector” and not “culprit”.

## 1. Introduction

Dementia is becoming a major health problem, with a large population affected by this disease in the world. According to statistics from the World Health Organization (WHO), more than 55 million people have dementia globally. Dementia includes vascular dementia, Alzheimer’s disease (AD), Lewy body disease (LBD), etc. [1]. Among small vascular diseases and AD, most of them were accompanied by chronic cerebral blood hypoperfusion and glucose hypometabolism, which could lead to cognitive/memory decline [2,3]. Reductions in cerebral blood flow are associated with early subjective cognitive decline, even in healthy older adults [4]. Therefore, chronic brain ischemia is a well-recognized risk factor in predisposing dementia.

Chronic cerebral hypoperfusion could lead to breakdown of the blood–brain barrier (BBB) and neurodegeneration [5]. As one part of the BBB, astrocyte and astrocyte-derived factors were proposed to drive BBB formation and maintenance, and also could regulate blood flow in and out of the CNS [6]. Astrocytes are the most abundant cell type in the brain, and they provide critical roles in CNS homeostasis. Astrocytes are involved in glutamate metabolism, glucose metabolism, Aβ degradation, and the regulation of neuron excitability, etc. [7]. Chronic hypoperfusion-induced ischemia and hypoxia could over-activate neuroinflammation, boost cytokines released by harmful activated microglia and aggravated neuroinflammation eventually [8]. It is well established that astrocytes are activated and actively participate in the above process [9]. Astrocytes have been found to divide into two phenotypes [10], in which one is the A1 cell type that plays an adverse role in the pathogenesis of AD [11], Huntington’s disease (HD) [12], Parkinson’s disease (PD) [13], etc. In AD, the activated microglia cells secrete some pro-inflammatory cytokines, which induce more astrocytes transformed into A1 type [11]. Currently, researchers believe that during an ischemic insult, astrocytes are activated promptly and could have both either a detrimental or a protective role depending on which type they turn into. Studies found that in cerebral ischemia and reperfusion, astrocytes transformed to the neurotoxic type A1 in the initial stage due to neuroinflammation and then into the neuroprotective A2 type, which could exert beneficial effects on CNS repair [14,15]. Therefore, it is imperative to know the phenotype transformation during chronic cerebral hypoperfusion and the underlying mechanism.

Here, we used a BCAO rat model to observe the phenotypes of astrocytes during chronic cerebral blood hypoperfusion. We found that astrocytes took on a protective role by transformation into type A2, and revealed some relevant signaling pathway possibly responsible for this protection.

## 2. Materials and Methods

### 2.1. Animals and Surgery

Male Sprague Dawley 8 weeks old rats (Vital-River, Beijing, China) were raised in the Experimental Animal Center of Capital Medical University under a 12:12 h light/dark cycle. The rats were randomly divided into BCAO group and sham-operated control group (7 rats per group). The detailed operations of BCAO surgery were as described previously [16]. The bilateral common carotid arteries were carefully separated and ligated with thin threads. The control group performed the same procedure, but the vessels were not ligated. After 4 weeks of ligation, the rats were used for experiments. All procedures involving animals complied with the guidelines of Ministry of Science and Technology of China and approved by the Animals Care and Use Committee at Capital Medical University (AEEI-2019-081, approval on 17 June 2019), Beijing, China.

### 2.2. Morris Water Maze Test

Morris water maze test was used to estimate spatial memory of rats, as previously described [17]. Rats were tested for 6 days consecutively in this test. In the first 2 days, the rats were trained to search the hidden platform. Each rat was placed facing the wall at the quadrant opposite to the platform. The rats who failed to find the platform within 120 s were placed on the platform for 10 s for reinforcement. Thereafter, in the following 3 days, the rats were evaluated to find the hidden platform, and the escape latency was recorded. On the sixth day, the platform was moved away, the number of target platform crossings and the time spent in the quadrant where the platform was previously placed were recorded.

### 2.3. Real-Time Quantitative PCR

Total mRNA was isolated from the parietal lobe of the rat brains, culture of rat cortical astrocytes, or C6 cells using an RNAsimple Total RNA Kit (TIANGEN, Beijing, China), and mRNA was reverse-transcribed using a FastQuant RT Kit (with gDNase) (TIANGEN, China) according to the manufacturer’s manual. Real-time Quantitative PCR was carried out with a ChamQ^TM^ SYBR qPCR Master Mix (Vazyme, Nanjing, China). The total reaction system was 20 µL, 95 °C 3 min, 40 cycles for 95 °C 10 s, 56 °C 30 s, and 72 °C 30 s. GAPDH and the average value of the controls were set as the internal parameter, and the relative mRNA levels were calculated with the 2^−ΔΔCt^ method. The primers are listed in Table 1.

### 2.4. Western Blot

Parietal lobes of rat cortexes, astrocytes of primary cultures, or C6 cells were lysed in RIPA lysate containing phosphatase inhibitor mixture and protease inhibitor cocktail (Applygen, Beijing, China). C6 cells were kindly provided by Professor Zhi-Qing David Xu. Supernatant proteins were separated using SDS-PAGE on 10%, 12%, or 15% gels and transferred onto PVDF membranes. After blocking with 5% non-fat milk in 1× Tris-buffered saline, pH7.5, containing 0.1% Tween 20 (TBST) for 2 h at room temperature, blots were incubated with primary antibodies in 1× TBST overnight at 4 °C. The primary antibodies included rabbit anti-S100a10 (1:1000), Connexin 43 (1:4000), Aquaporin 4 (1:1000) antibody (Proteintech, Rosemont, IL, USA), mouse anti-GFAP (1:5000), GAPDH (1:5000), β-actin (1:5000) antibody (Proteintech), mouse anti-C3 (1:200), Apolipoprotein E (1:200) (Santa, Dallas, TX, USA), and rabbit anti-phospho-ERK (1:1000), ERK (1:1000), phospho-JNK (1:1000), JNK (1:1000), phospho-AKT (1:1000), and AKT (1:1000) (Cell Signaling, Boston, MA, USA). Then, blots were washed with 1× TBST three times and incubated with horseradish peroxidase-conjugated secondary antibody (goat anti-rabbit or goat anti-mouse) (Jackson, West Grove, PA, USA) at RT for 2 h. Finally, the blots were rinsed and visualized using SuperEnhanced chemiluminescence detection reagents (Applygen) according to the manufacturer’s instructions. Optical densities of individual blot were quantified using Image J software (version 1.38). Ratios of target antibody to GAPDH or β-actin were calculated for each sample, and fold-changes were shown compared to the control.

### 2.5. The Enzyme-Linked Immunosorbent Assay (ELISA)

Parietal cortical tissues of rats were homogenized and sonicated in cold PBS containing a protease inhibitor cocktail (Applygen, China), centrifuged for 10 min at 5000× *g*, and then the supernate was assayed according to the manufacturer’s manual from the Enzyme-linked Immunosorbent Assay Kit for Complement Component 3 (C3) (Cloud-Clone, Wuhan, China) or for S100 Calcium-Binding Protein A10 (S100A10) (Mlbio, Shanghai, China).

### 2.6. Primary Culture of Rat Cortex Astrocytes

The cultures of rat cortex astrocytes were prepared from the brain of 1-day-old neonatal Sprague Dawley rats. The brainstem, cerebellum, and diencephalons were removed in cold PBS buffer and then the meninges were peeled off. The cortex was minced using scissors, incubated with 0.25% trypsin–EDTA at 37 °C for 5 min, and filtered through a 200 mesh strainer. After incubating at 37 °C in 5% CO_2_ for 1 h, the culture mediums were collected and re-suspended in DMEM/F12 supplemented with 10% fetal bovine serum, 1% penicillin/streptomycin and plated on dishes at 37 °C in 5% CO_2_. About 2 weeks later, the cultures reached confluence and were shaken at 250 rpm for 18 h at 37 °C to dislodge cells adhering to the astrocyte layer. The confluent cultures were trypsinized and sub-plated onto dishes. In this present study, astrocytes were used at passage 3. When astrocytes were transformed, cobalt chloride, fibrinogen (Merck, Rahway, NJ, USA), or low-glucose DMEM was added in the medium.

### 2.7. Cytotoxicity Assay

Sensitivities of astrocytes or C6 cells to various chemicals were examined using the Cell counting Kit-8 (CCK-8, Applygen). Cells were plated in 96-well plates, incubated at 37 °C in 5% CO_2_ for 24 h, culture medium were replaced with new medium and drugs, and incubated for an additional 24  h. In total, 10 μL CCK-8 reagent was added into each well and incubated at 37 °C in 5% CO_2_ for 2 h before reading at a wavelength of 450 nm. Absorbances were converted to percentages for comparison with the vehicle group.

### 2.8. Immunofluorescence

Astrocyte cultures on poly-L lysine-coated glass coverslips were rinsed twice with 0.01M PBS, pH 7.2–7.4, and fixed with 4% paraformaldehyde for 15 min at room temperature (RT). The cells were rinsed three times with 0.01M PBS, incubated with 0.01M PBS containing 0.3% Triton X-100 for 30 min, blocked in 10% normal goat serum for 1 h, incubated with primary monoclonal anti-GFAP mouse antibody (1:350) (Sigma, St. Louis, MO, USA) overnight at 4 °C. The cells were rinsed three times with PBS, incubated with secondary goat anti-mouse Alexa Fluor 488 (Invitrogen, Waltham, MA, USA) for 2 h at RT, rinsed three times with 0.01M PBS, mounted with anti-fade mounting solution, and observed under a confocal microscope (Leica, Teaneck, NJ, USA).

### 2.9. Statistical Analysis

Results were presented as means ± SEM. Data were evaluated using ANOVA or Student’s *t*-test. A value of *p* < 0.05 was considered statistically significant.

## 3. Results

### 3.1. Artery Occlusion Caused Significant Memory and Cognitive Function Deficit

**Figure 1:**
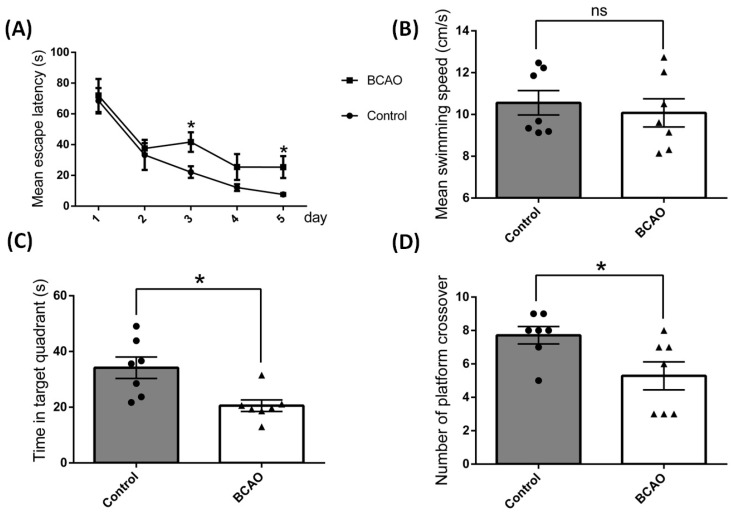
Cognitive performance tested using a Morris Water Maze in bilateral common carotid artery occlusion (BCAO) rats after 4 weeks of ligation. The rats were tested at a designed time for five consecutive days. (**A**) Mean escape latency, (**B**) mean swimming speed, (**C**) time in target quadrant, (**D**) number of platform crossover measured. Data were expressed as mean ± SEM, * *p* < 0.05 versus the control rats; ns, no significance (*n* = 7).

In order to determine the effects of chronic ischemia and hypoxia on rat brain cognitive function, a Morris Water Maze test was performed among the BCAO rats and their counterpart control rats. As shown in Figure 1, after ligation for 4 weeks, the BCAO rats showed higher average escape latency from day 3 to day 5 (Figure 1A), although there were no differences in the average swimming speed of rats between these two groups (Figure 1B). More importantly, the BCAO rats demonstrated significantly less time and number crossing in the target quadrant where the platform was previously established on day 6 of the memory retrieval test (Figure 1C,D). These results consistently imply that artery occlusion caused significantly memory and cognitive deterioration in these animals.

### 3.2. Artery Occlusion Inhibited the Gene Expression of Marker Proteins of Both Types of Astrocyte, but Increased the Protein Level of S100a10

**Figure 2:**
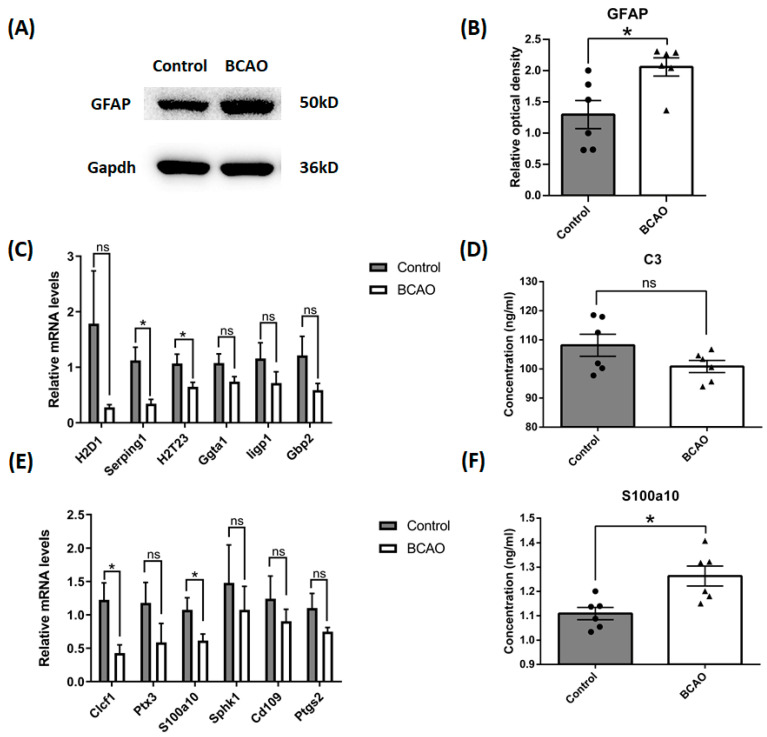
Reactive astrocyte phenotype in the cortex of BCAO rats after 4 weeks of ligation. (**A**,**B**) The protein levels of GFAP were tested using Western blot, and the data were normalized to Gapdh blot. (**C**) A1 reactive astrocyte phenotypes were tested using Real-time Quantitative PCR. (**D**) C3 protein concentration was tested using Elisa. (**E**) A2 reactive astrocyte phenotypes were tested using QPCR. (**F**) S100a10 protein concentration was tested using Elisa. Data were expressed as mean ± SEM, * *p* < 0.05 versus the control rats; ns, no significance (*n* = 6).

Astrocyte reactivation in ischemic insults has been reported in a previous study [18]. In order to validate the cellular level changes of astrocytes in our BCAO rats, we employed a Western blot assay to study the reactivation of astrocytes after 4 weeks of ligation. We found that the protein content of GFAP was up-regulated significantly in the BCAO rat cortex (Figure 2A,B). To further elaborate the characteristics of these reactivated astrocytes, phenotype markers for both A1- and A2-reactive astrocytes were measured with Real-time Quantitative PCR and ELISA assays. Using Real-time Quantitative PCR, we found that mRNA transcripts of both A1 type and A2 type astrocyte markers were simultaneously significantly down-regulated, including serpin family G member 1 (Serping1) and RT1 class Ib (H2T23) for A1 phenotype (Figure 2C) and cardiotrophin-like cytokine factor 1 (Clcf1) and S100 calcium-binding protein A10 (S100a10) for A2 phenotype (Figure 2E) after 4 weeks of ligation. Interestingly, on the protein level, we found that the marker protein of the A2 phenotype, S100a10, was significantly up-regulated in the BCAO rat cortex, while the content of C3 in the A1 type marker protein showed an obvious reducing trend, albeit no significant difference (Figure 2D,F), with ELISA analysis after 4 weeks of ligation. These results suggest that astrocytes in the present model were promoted to A2 phenotypes.

### 3.3. Artery Occlusion Inhibited the Protein Expression of CX43, Not AQP4 and APOE

**Figure 3:**
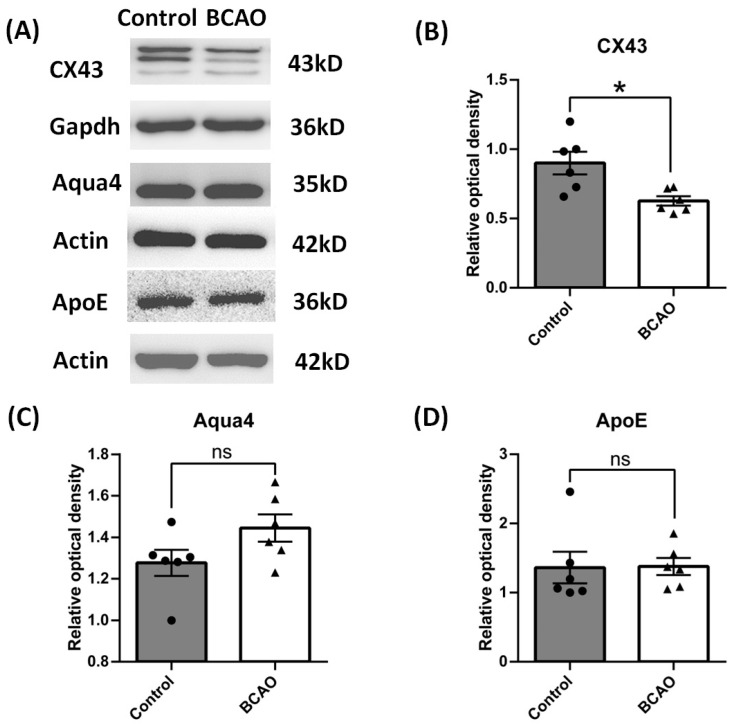
Reactive astrocyte function in the cortex of BCAO rats after 4 weeks of ligation. (**A**) Representative blots of CX43, Aqua4, and ApoE. (**B**–**D**) Ratios of CX43, Aqua4, and ApoE to Gapdh or Actin were calculated and compared. Data were expressed as mean ± SEM, * *p* < 0.05 versus the control rats; ns, no significance (*n* = 6).

Besides the phenotype change in astrocytes, we further used Western blot to study the function proteins of astrocytes especially related to the blood–brain barrier. Connexin 43 (CX43), aquaporin 4 (AQP4), and apolipoprotein E (APOE) are generally considered to be astrocyte-derived proteins and are closely associated with pathogenesis of ischemia or stroke [19,20,21]. We found that after 4 weeks of chronic ischemia and hypoxia, the expression of CX43 was significantly down-regulated; however, the levels of AQP4 and APOE expression had no change compared to the control group (Figure 3).

### 3.4. In Vitro Hypoxia Inhibited the Gene Expression of Marker Protein of Both Types of Astrocyte, but Increased the Protein Level of S100a10

**Figure 4:**
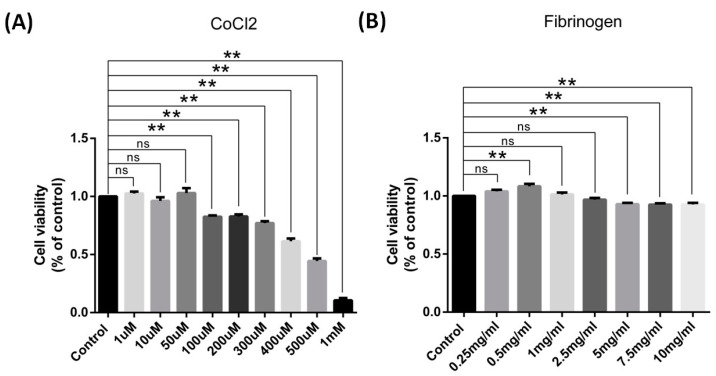
Cobalt chloride and fibrinogen induced toxicity in cultured cortical astrocytes of rats for 24 h. (**A**,**B**) Cell viability was determined using a CCK assay. (**A**) CoCl2 caused a dose-dependent effect on astrocyte viability (*n* = 3). (**B**) The effect of fibrinogen was not concentration-dependent or prominent (*n* = 9). Data were expressed as mean ± SEM, ** *p* < 0.01 versus the control group; ns, no significance.

To further confirm the findings of the in vivo studies and illustrate the possible underlying mechanisms relevant to the changes, we employed in vitro cell culture models of astrocytes by treating them with cobalt chloride (CoCl2) to mimic the ischemic insults in culture conditions [22]. Brain insults and astrocyte phenotype changes have been found to be closely related to BBB dysfunction, which could be reflected in a culture model by using fibrinogen to stimulate the cells [23]. In these culture models, the toxicity of cobalt chloride and fibrinogen to astrocytes were tested using a CCK-8 kit first, which used a formazancon centration produced by vital cell numbers to reflect the toxicity of substance. The results show that the toxicity of CoCl2 was dose-dependent. In total, 100 μM CoCl2 caused 17% astrocytes to die compared to the control group, while 300 μM CoCl2 caused 23% astrocytes to die, and only 10% astrocytes survived at 1 mM CoCl2 treatment (Figure 4A). Meanwhile, we also found that the effects of fibrinogen were not dose-dependent. Low concentrations of fibrinogen, such as 0.5 mg/mL, caused astrocyte hyperplasia, about 8% higher than the control group, while high concentrations of fibrinogen, such as 5 mg/mL and 10 mg/mL, caused 7% astrocytes to die (Figure 4B). Therefore, 250 μM CoCl2, 0.5 mg/mL, and 5 mg/mL fibrinogen were used in the following studies.

**Figure 5:**
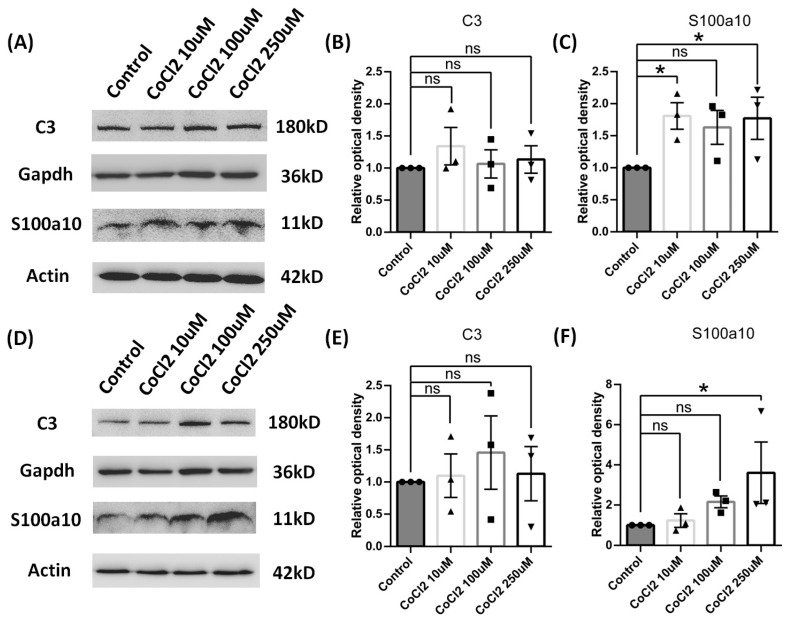
Reactive phenotype in cultured cortical astrocytes of rats by different concentrations of CoCl2 and time duration. (**A**–**C**) After 24 h, the protein levels of C3 and S100a10 were tested using Western blot, and the data were normalized to Gapdh or Actin blot. (**D**–**F**) After 96 h, the protein levels of C3 and S100a10 were tested using Western blot, and the data were normalized to Gapdh or Actin blot. Data are expressed as mean ± SEM, * *p* < 0.05 versus the control group; ns, no significance (*n* = 3).

**Figure 6:**
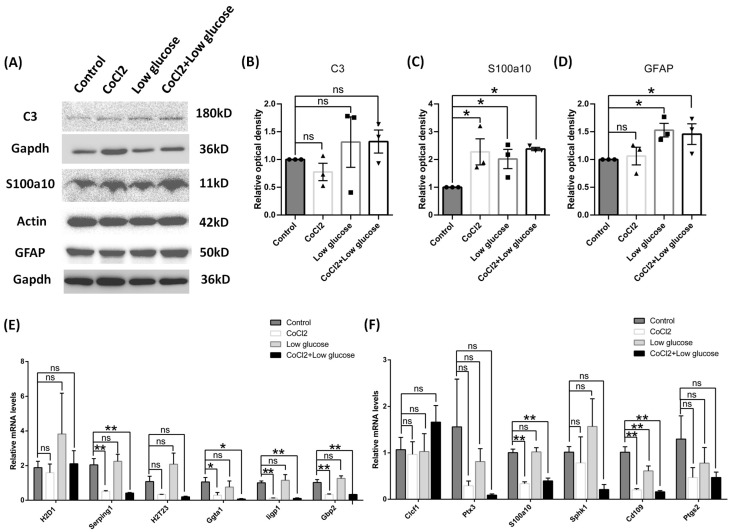
Reactive phenotype in cultured cortical astrocytes of rats by cobalt chloride and low glucose for 24 h. (**A**–**D**) The protein levels of C3, S100a10, and GFAP were tested using Western blot and the data were normalized to Gapdh or Actin blot. (**E**,**F**) A1 and A2 reactive astrocyte phenotypes were tested using QPCR. Data were expressed as mean ± SEM, * *p* < 0.05,** *p* < 0.01 versus the control group; ns, no significance (*n* = 3).

**Figure 7:**
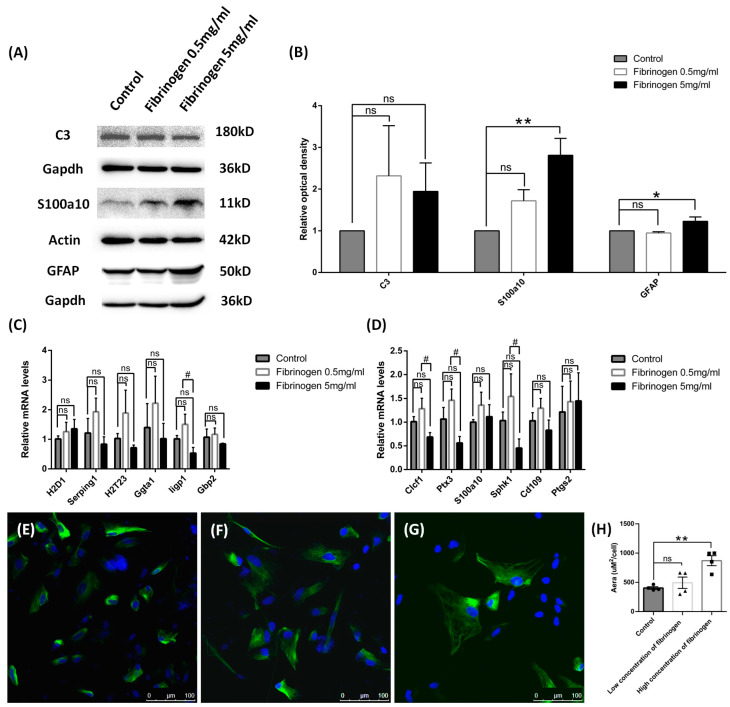
Reactive phenotype in cultured cortical astrocytes of rats by fibrinogen for 24 h. (**A**,**B**) The protein levels of C3, S100a10, and GFAP were tested using Western blot, and the data were normalized to Gapdh or Actin blot. (**C**,**D**) A1 and A2 reactive astrocyte phenotypes were tested using QPCR. (**E**–**G**) Immunofluorescence staining of GFAP using control (**E**). Low concentration of fibrinogen and (**F**) high concentration of fibrinogen (**G**). (**H**) Analysis of cell area in immunofluorescence staining of GFAP. Data are expressed as mean ± SEM, * *p* < 0.05, ** *p* < 0.01 versus the control group; # *p* < 0.05, high concentration versus low concentration of fibrinogen group; ns, no significance (*n* = 3).

Cultured astrocytes were used to mimic the hypoxia condition via cobalt chloride treatment at different time of points. In total, 10 μM, 100 μM, or 250 μM of cobalt chloride caused increased S100a10 expression level at both 24 h (Figure 5C) and 96 h (Figure 5F), and 250 μM CoCl2 had the most significant difference. But the C3 protein expression was not significantly changed in all the doses (Figure 5B,E), which made 250 μM CoCl2 the favorable condition for mechanism study since it had a similar protein expression pattern to the in vivo study. Therefore, 250 μM CoCl2 and 24 h were used in the culture model for further tests to match the results from the in vivo studies. In chronic hypoperfusion of cerebral blood flow, both hypoxia and low glucose usually happened simultaneously. After 250 μM cobalt chloride, 500 mg/l glucose or both were given to astrocyte culture for 24 h [24]. As expected, astrocytes under these stimulates also turned out to be A2 reactive astrocyte instead of A1. S100a10 protein concentration was increased in all conditions (cobalt chloride or low glucose alone or together) (Figure 6C), but there was no change in C3 protein level (Figure 6B). We also found that both mRNA of A1 and A2 transcripts were down-regulated, such as Serping1, glycoprotein alpha-galactosyltransferase 1 (Ggta1), similar to interferon-inducible GTPase (Iigp1), and guanylate binding protein 2 (Gbp2) for A1 phenotype (Figure 6E), as well as S100a10 and CD109 molecule (Cd109) for the A2 phenotype (Figure 6F), especially in hypoxia conditions for 24 h. BBB disruption is usually subsequent to the hypoperfusion of cerebral blood flow, such as stroke along with leakage of blood components, including fibrinogen, into the brain parenchyma, leading to immune and neuroinflammatory responses [25]. In our study, astrocytes in cultures were then given 0.5 mg/mL or 5 mg/mL fibrinogen for 24 h. Similar to the results of hypoxia and low glucose (see Supplementary Materials Figure S1), compared to the control group (Figure 7E), the morphology of astrocyte had no obvious change in the low-concentration fibrinogen group (Figure 7F), but high-concentration fibrinogen caused astrocyte hypertrophy (Figure 7G,H), and S100a10 protein concentration elevated, but C3 had no changes (Figure 7B). mRNA of A1 transcripts such as Iigp1, and mRNA of A2 transcripts such as Clcf1, pentraxin 3 (Ptx3), and sphingosine kinase 1 (Sphk1) were down-regulated, especially in the 5 mg/mL fibrinogen group compared to the low concentration of fibrinogen group (Figure 7C,D) for 24 h. The results in vitro models were partly consistent with in vivo models, both suggesting that the A2 phenotype was the predominant type.

### 3.5. In Vitro Hypoxia Inhibited the Expression of CX43 Protein, Not AQP4 and APOE, in Primary Cultures of Astrocytes and C6 Cell Lines

**Figure 8:**
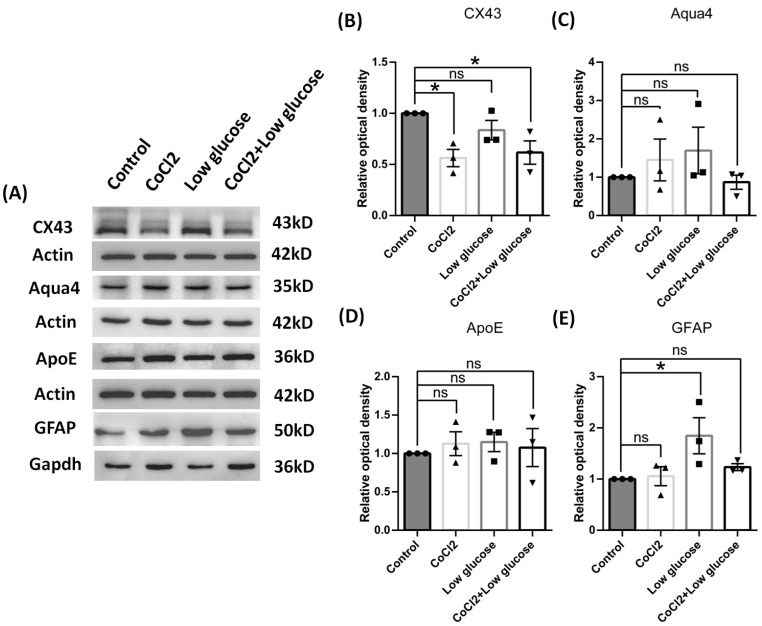
Function of reactive cultured cortical astrocytes of rats by cobalt chloride and low glucose for 72 h. (**A**) Representative blots of CX43, Aqua4, ApoE, and GFAP. (**B**–**E**) Ratios of CX43, Aqua4, ApoE, and GFAP to Actin or Gapdh were calculated and compared. Data were expressed as mean ± SEM, * *p* < 0.05 versus the control group; ns, no significance (*n* = 3).

Next, we explored whether the expression levels were changed among proteins related with astrocyte function in the culture models by measuring CX43, aquaporin 4, and aolipoprotein E. After under the chronic condition of hypoxia and low glucose for 72 h, cultured astrocyte still could maintain reactivation featured with elevated GFAP expression. We found the expression of CX43 was significantly down-regulated in the model cells, but the levels of aquaporin 4 and aolipoprotein E showed no significant changes compared to the control group, which sit well with our results from the in vivo model (Figure 8).

**Figure 9:**
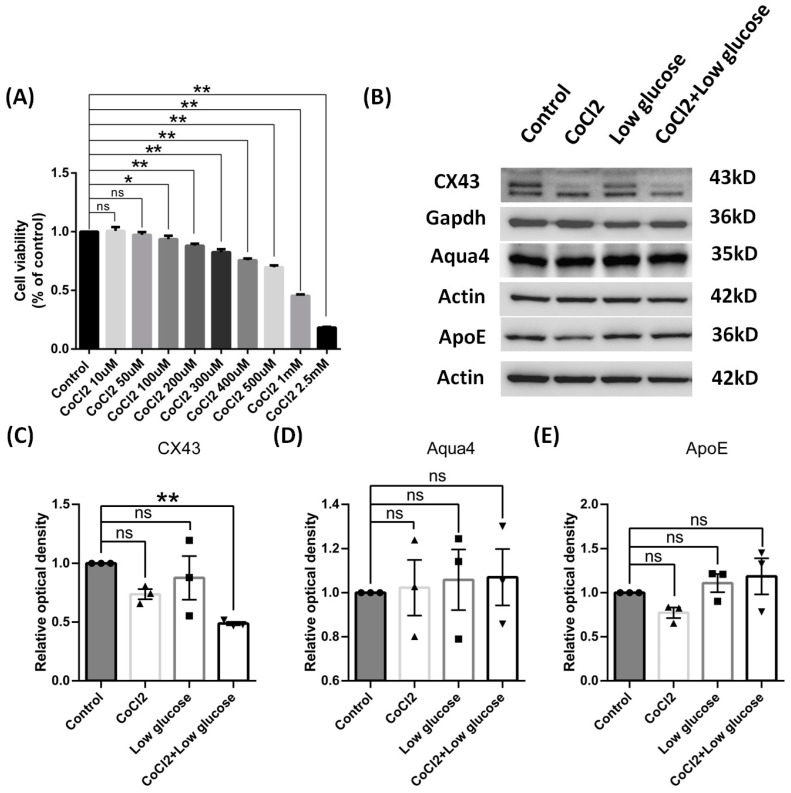
Effects of C6 cells by cobalt chloride or low glucose for 24 h. (**A**) Cell viability was determined using a CCK assay, and CoCl2 caused a dose-dependent effect on C6 cell viability. (**B**) Representative blots of CX43, Aqua4, and ApoE. (**C**–**E**) Ratios of CX43, Aqua4, and ApoE to Gapdh or Actin were calculated and compared. Data were expressed as mean ± SEM, * *p* < 0.05, ** *p* < 0.01 versus the control group; ns, no significance (*n* = 3).

We also used astrocyte cell line C6 to further validate the effects of hypoxia and low glucose. The toxicity effects of cobalt chloride on C6 cells were similar with those from astrocyte cultures (Figure 9A), and, therefore, 250 μM CoCl2 concentration was also used as before. After undergoing hypoxia and low glucose challenges for 24 h, theses C6 cells expressed significant lower level of CX43 as the primary astrocyte cultures (Figure 9C), but comparable levels of aquaporin 4 and apolipoprotein E to the control group, which was also consistent with the results from astrocyte cultures (Figure 9D,E).

### 3.6. In Vitro Hypoxia Inhibited the Phosphoraltion of ERK and JNK Not AKT

**Figure 10:**
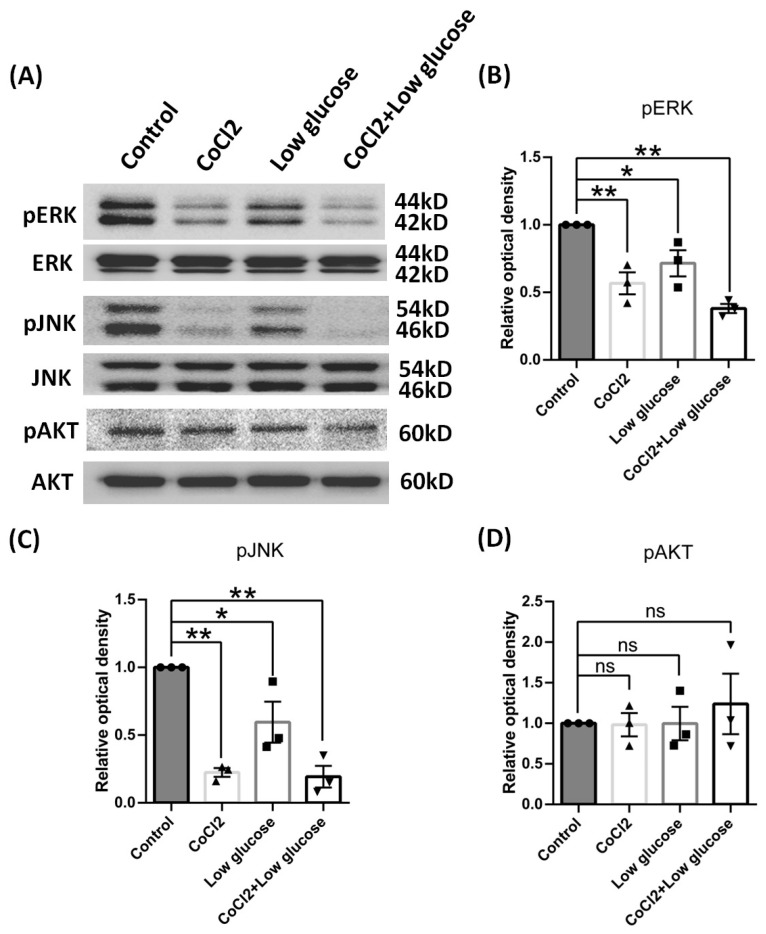
Signal pathway proteins of C6 cells by cobalt chloride or low glucose for 24 h. (**A**) Representative blots of pERK, pJNK, pAKT. (**B**–**D**) Ratios of pERK, pJNK, pAKT to ERK, JNK, and AKT were calculated and compared. Data were expressed as mean ± SEM, * *p* < 0.05, ** *p* < 0.01 versus the control group; ns, no significance (*n* = 3).

CX43 expression reportedly could be regulated by the MAPK/ERK and PI3K–pAKT signaling pathways [26,27]. Therefore, we also carried out Western blot assays to investigate if these signaling pathways were possibly involved in the CX43 expression changes under our culture models. We found that pERK and pJNK in C6 cells were significantly down-regulated after hypoxia and low glucose exposure, while pAKT protein expression was not affected by these insults (Figure 10). Also pSTAT3 signal pathway was not affected, but pCREB was activated by cobalt chloride alone (see Supplementary Materials Figure S2). Therefore, we could claim that the reduced expression of CX43 might be attributed to the suppression of pERK and pJNK due to ischemic insults.

## 4. Discussion

In many neural system pathologies, such as trauma, stroke, and neurodegenerative diseases, astrocytes activation is quite often, namely as “astroglisosis”. During the activation, the cell bodies and cellular processes of astrocytes become hypertrophied, and intermediate filament protein, particularly glial fibrillary acidic protein (GFAP), are up-regulated [28,29]. Reactive astrocytes have been classified into two phenotypes: A1 type (neurotoxic) and A2 type (neuroprotective) [10]. In the pathogenesis of any CNS disease, the different factors involved may lead to the different preferring polarizations of astrocytes [30].

Reactive astrocytes are triggered during acute ischemic insult, but the reactive profile during chronic ischemia remains elusive. Under chronic cerebral hypoperfusion condition, A1 astrocytes were the predominant astrocyte type in the white matter [31,32]. However, in a study of astrocytes derived from hippocampus, the transcripts of A1 type were down-regulated in chronic cerebral hypoperfusion mice [33]. In our study, BCAO rats were used to mimic chronic brain ischemia in vivo. In chronic hypoperfusion of cerebral blood flow, both hypoxia and low glucose usually happened simultaneously, and BBB disruption is usually subsequent to the hypoperfusion, such as leakage of blood components, so CoCl2, low glucose, and fibrinogen were used in in vitro models. In all the models, S100a10 was significantly up-regulated, which meant the astrocytes were likely transformed into an A2 phenotype in different types of nerve cell insults. S100a10 was a member of the S100 EF-hand protein family, which connected certain membrane proteins with annexin A2 as a bridge [34,35]. Studies show that S100a10 could negatively regulate TLR signaling pathways in macrophages, suggesting an important role in regulating inflammatory response [36]. S100a10 was also found in astrocytes [37]. Up-regulation of S100a10 may promote the survival of neurons through secretion of neuroprotective factors in astrocytes upon MPTP exposure [38]. The up-regulation of S100a10 may come from A1 phenotype reactive astrocyte transformation, or even from ependymal cells [39].

Furthermore, a single indicator, such as S100a10, only showed part of an integrated effect, and studies have found that the functions of astrocytes are complicated. During brain injury, reactive astrocytes could re-uptake glutamate from synapses cleft, preventing excitotoxicity to neurons, but also were capable of producing pro-inflammatory cytokines and matrix metalloproteinase (MMP), which could cause subsequent BBB disruption [40]. Although glial scars, mainly formed by reactive astrocytes, were thought to act as a physical barrier to encapsulate damaged tissue, they also had an inhibitory effect on axonal regrowth [41]. In mice after transient focal cerebral ischemia, astrocytes could transfer mitochondria to adjacent neurons to help neuron survival [42]. And in a mouse model of focal cerebral ischemia, astrocytes trans-differentiated to neural progenitor cells to help CNS repair [43]. Mostly in the acute phase of stroke, astrocytes could limit tissue damage, but in the chronic phase, reactive gliosis seems to inhibit neural axonal sprouting [44]. Astrocytes also took a role of astrogliosis with pro-inflammatory phenotypes in AD, PD, and HD [45]. Even in a healthy condition, astrocytes have different molecular expression pattern in different regions of the brain [46]. In our study, we also found that inducible nitric oxide synthase (iNOS) was down-regulated and Glutamate cysteine ligase catalytic subunit (GCLC) was up-regulated in cultured astrocytes (see Supplementary Materials Figure S3), which suggests that astrocytes took on a protective role.

After a long time of cerebral hypoperfusion in vivo, and low oxygen and glucose in astrocytes or C6 cells in vitro, we also found that CX43 was down-regulated, but no changes in AQP4 and APOE expression were found on protein levels. Aquaporin 4 (AQP4) is primarily expressed in astrocyte foot processes, which are involved in water movement, clearance of brain metabolic waste, cell migration, and neuroexcitation [47,48]. AQP4 concentration and polarization were changed in the acute phase of cerebral chronic hypoperfusion, but in the chronic phase, it recovered to normal status [49,50]. These results sat well with our findings and further supported the establishment of our chronic hypoxia models.

Apolipoprotein E (APOE) is expressed richly in astrocyte cell processes and could be secreted by astrocytes [51]. The APOE gene variant is the strongest genetic risk factor for AD. APOE plays an important role in Aβ clearance, phosphorylation of tau, and neuroinflammation enhancement [52]. APOE-deficient mice exerted more neural death and decreased white matter integrity and cognitive function after chronic cerebral hypoperfusion, which suggests a protect effect of APOE in ischemia [53]. APOE level was found to be elevated in synaptic protein under chronic cerebral hypoperfusion [54]. In our study, APOE did not have significant changes in our chronic hypoxia models, which might suggest that APOE did not play a role in chronic hypoxia injury.

Connexin 43 (CX43), as the major astrocytic gap junction protein, is expressed mainly in astrocytes in vivo and in vitro, and it is critical for buffering K^+^, regulation of cell migration, proliferation, and survival [55]. The surface and mitochondrial expression of CX43 under chronic cerebral hypoperfusion were increased in rats [56]. But inhibition of CX43 protected myelin integrity and rescued cognitive decline in the chronic cerebral hypoperfusion mice [57]. The expression change in Cx43 after ischemic stroke remained debated due to inconsistent reports from different labs [58]. It had been well known that Cx channels, including Cx43, can facilitate the transfer of toxic signals from dying cells to healthy neighbors under certain pathologic conditions [59]. In our data, the down-regulation of CX43 in the brain cortexes, astrocyte cultures, and C6 cells may be an adaptive response to both hypoxia and low glucose. Reduced-expression Cx43 were equal to “close” the channels between astrocyte–astrocyte and other cells, which could lead to de-amplifying of injury spreading. This also was consistent with the fact that astrocytes turned to the A2 phenotype under hypoperfusion conditions in the in vivo and in vitro models in the present study because of the neuroprotective role of this A2 type of astrocyte. A previous study has shown that CX43 has three bands on SDS PAGE assays, which include the phosphorylated state of CX43, referred to as P1 and P2, and non-phosphorylated CX43, referred to as P0 [60]. In our data, all of the densities of the three bands decreased, especially the phosphorylation bands in BCAO rats and C6 cells, which was in line with a previous report [61]. Since ERK1/2 had been found to modulate phosphorylation of CX43 [26], JNK inhibitors decreased the expression of phosphorylation of CX43 in C6 cells [27]. Under hypoxia and low glucose in our models, we found that the phosphorylation of ERK1/2 and JNK were decreased. And, therefore, the down-regulation of phosphorylated CX43 might be caused by the inhibition of ERK1/2 and JNK due to hypoxia injury. Since glutamate could be released from astrocytes via CX43 [62], the down-regulation of CX43 may decrease the injury to neurons by mitigating the spreading of glutamate toxicity. A study also demonstrated that inhibiting CX43 degradation could transit astrocytes to anti-inflammatory status during ischemic stress [63], which was consistent with our findings here of astrocytes turning into the A2 type instead of the A1 type. But more data from genetically modified animal models are required from future studies with knocking in or knocking down strategies to further validate this hypothesis. Based on the data from the present study, we hypothesize that Cx43 could be one of the key players in CNS during ischemia.

## 5. Conclusions

Our data suggest that in the pathological condition of chronic cerebral hypofusion, astrocytes could tend to preferably transform themselves more to the neuroprotective type A2 instead of A1. CX43 protein was also down-regulated in ischemic stress, which might be attributed to the inhibition of MAPK/ERK and PI3K–pAKT signaling pathways. These findings might lay some of the foundations for subsequent research on the roles of CX43 in ischemic insults and how to leverage this protein to better study the hypoxia injury in the CNS.

## Figures and Tables

**Table 1:** List of qPCR primers.

Primer Name	Primer Sequence
H2D1	forward: ATGGAACCTTCCAGAAGTGGG
	reverse: GAAGTAAGTTGGAGTCGGTGGA
Serping1	forward: TGGCTCAGAGGCTAACTGGC
	reverse: GAATCTGAGAAGGCTCTATCCCCA
H2T23	forward:ATTGGAGCTGTTGTGAGGAGG
	reverse: CCACGAGGCAACTGTCTTTTC
Ggta1	forward: TCTCAGGATCTGGGAGTTGGA
	reverse: GAGTTCTATGGAGCTCCCGC
Iigp1	forward: ATTTGGCTCGAAGCCTTTGC
	reverse: ACGGCATTTGCCAGTCCTTA
Gbp2	forward: TAAAGGTCCGAGGCCCAAAC
	reverse: AACATATGTGGCTGGGCGAA
Clcf1	forward: GACTCGTGGGGGATGTTAGC
	reverse: CCCCAGGTAGTTCAGGTAGGT
Ptx3	forward: CATCCCGTTCAGGCTTTGGA
	reverse: CACAGGGAAAGAAGCGAGGT
S100a10	forward: GAAAGGGAGTTCCCTGGGTT
	reverse: CCCACTTTTCCATCTCGGCA
Sphk1	forward: AAAGCGAGACCCTGTTCCAG
	reverse: CAGTCTGCTGGTTGCATAGC
Cd109	forward: GTCGCTCACAGGTACCTCAA
	reverse: CTGTGAAGTTGAGCGTTGGC
Ptgs2	forward: CTCAGCCATGCAGCAAATCC
	reverse: GGGTGGGCTTCAGCAGTAAT
GAPDH	forward: GACCACCCAGCCCAGCAAGG
	reverse: TCCCCAGGCCCCTCCTGTTG

## Data Availability

All data and materials used are available from the corresponding author upon reasonable request.

## Supplementary Materials

The following supporting information can be downloaded at: https://www.mdpi.com/article/10.3390/brainsci14121256/s1. Figure S1. Immunofluorescence staining of GFAP in cultured cortical astrocytes of rats by cobalt chloride and low glucose for 24 h; Figure S2. Signal pathway proteins of C6 cells by cobalt chloride or low glucose for 24 h; Figure S3. Reactive function in cultured cortical astrocytes of rats by cobalt chloride and low glucose for 72 h.
