# Supplementary material for: The Phenotype Changes of Astrocyte During Different Ischemia Conditions
Source: Brain Sci. 2024 Dec 14;14(12):1256. doi: 10.3390/brainsci14121256 (PMC11674399; doi:10.3390/brainsci14121256)
Supplement: Supplementary file 1 [file brainsci-14-01256-s001.zip › brainsci-3301745-supplementary.pdf]

Supplementary Materials

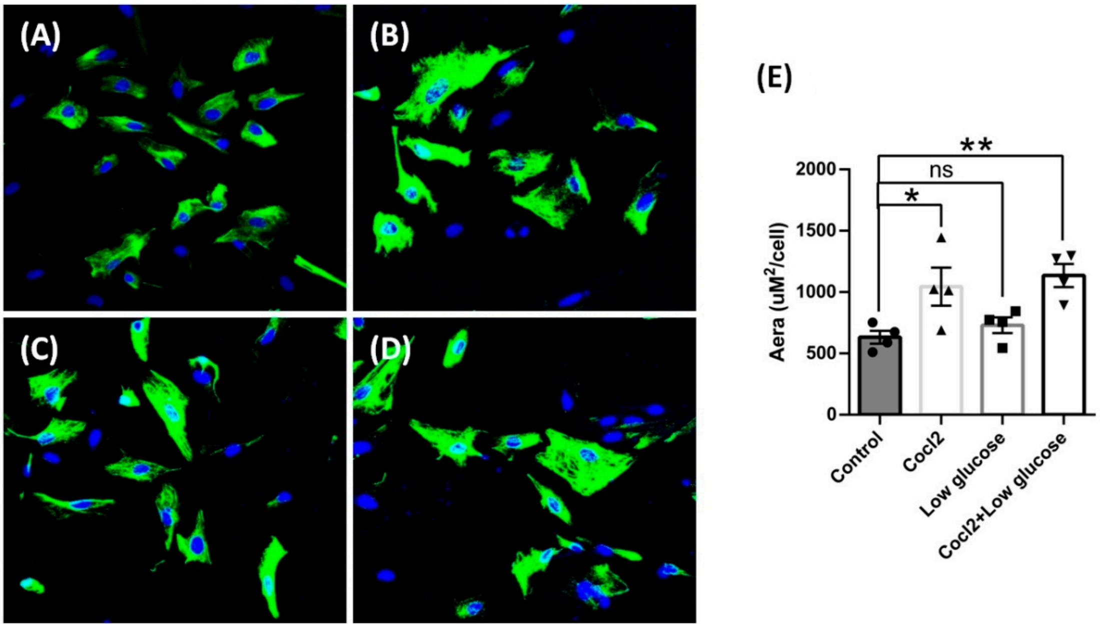

Figure S1. Immunofluorescence staining of GFAP in cultured cortical astrocytes of rats by cobalt chloride and low glucose for 24 h. (A) Control, (B) Cocl2, (C) Low glucose, (D) Cocl2 and low glucose. (E) Analysis of cell area in immunofluorescence staining of GFAP. Data were expressed as mean±SEM, *p<0.05, **p<0.01 versus the control group; ns, no significance(n=4).

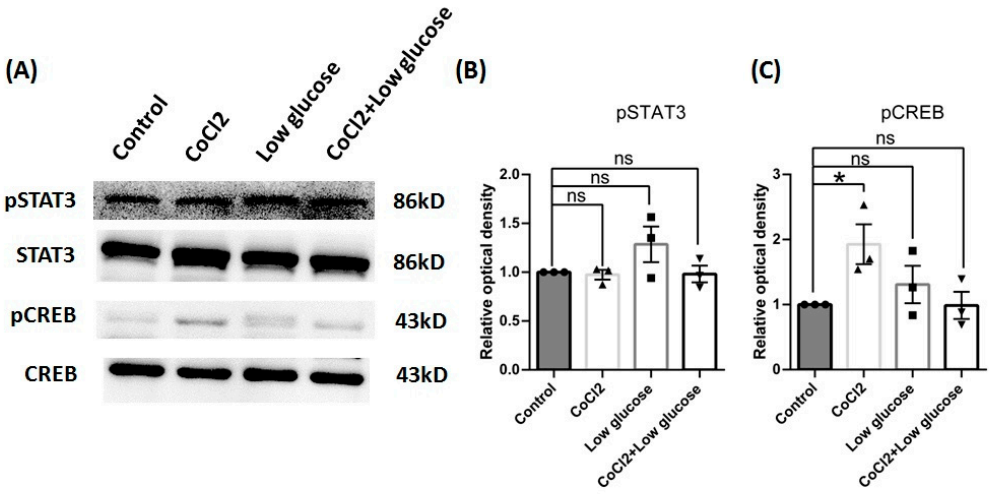

Figure S2. Signal pathway proteins of C6 cells by cobalt chloride or low glucose for 24 h. (A) Representative blots of pSTAT3, pCREB. (B),(C) Ratios of pSTAT3, pCREB to STAT3, CREB were calculated and compared. Data were expressed as mean±SEM, *p<0.05 versus the control group; ns, no significance (n=3).

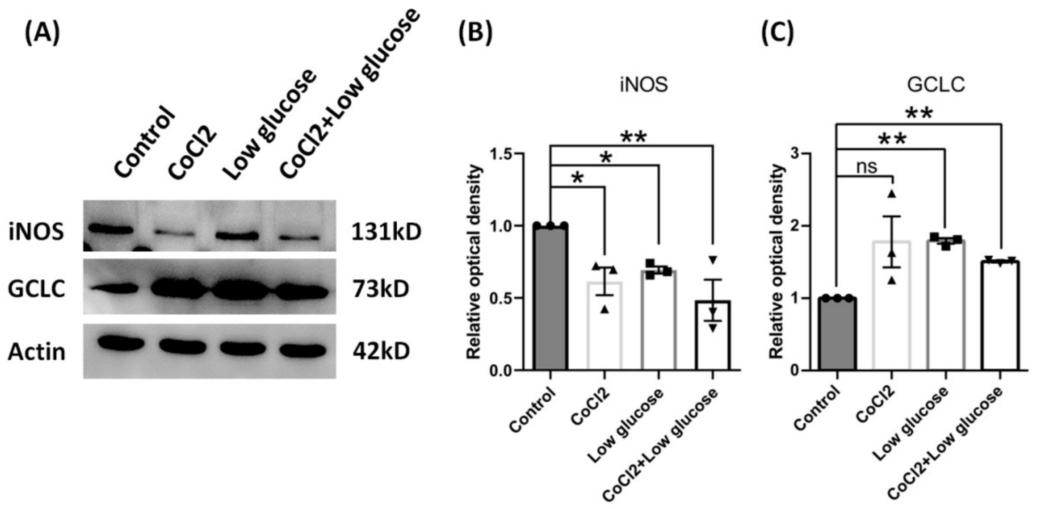

Figure S3. Reactive function in cultured cortical astrocytes of rats by cobalt chloride and low glucose for 72 h. (A) Representative blots of iNOS, GCLC. (B),(C) Ratios of iNOS, GCLC to Actin were calculated and compared. Data were expressed as mean±SEM, *p<0.05,**p<0.01 versus the control group; ns, no significance (n=3).
